# Effect of Peripheral 5-HT on Glucose and Lipid Metabolism in Wether Sheep

**DOI:** 10.1371/journal.pone.0088058

**Published:** 2014-02-04

**Authors:** Hitoshi Watanabe, Ryo Saito, Tatsuya Nakano, Hideyuki Takahashi, Yu Takahashi, Keisuke Sumiyoshi, Katsuyoshi Sato, Xiangning Chen, Natsumi Okada, Shunsuke Iwasaki, Dian W. Harjanti, Natsumi Sekiguchi, Hiroaki Sano, Haruki Kitazawa, Michael T. Rose, Shyuichi Ohwada, Kouichi Watanabe, Hisashi Aso

**Affiliations:** 1 Cellar Biology Laboratory, Graduate School of Agricultural Science, Tohoku University, Sendai, Japan; 2 Department of Animal Sciences, Faculty of Agriculture, Iwate University, Morioka, Japan; 3 Laboratory of Food and Biomolecular Science, Graduate School of Agricultural Science, Tohoku University, Sendai, Japan; 4 Institute of Biological, Environmental and Rural Sciences, Aberystwyth University, Cardiganshire, United Kingdom; University of Rouen, France, France

## Abstract

In mice, peripheral 5-HT induces an increase in the plasma concentrations of glucose, insulin and bile acids, and a decrease in plasma triglyceride, NEFA and cholesterol concentrations. However, given the unique characteristics of the metabolism of ruminants relative to monogastric animals, the physiological role of peripheral 5-HT on glucose and lipid metabolism in sheep remains to be established. Therefore, in this study, we investigated the effect of 5-HT on the circulating concentrations of metabolites and insulin using five 5-HT receptor (5HTR) antagonists in sheep. After fasting for 24 h, sheep were intravenously injected with 5-HT, following which-, plasma glucose, insulin, triglyceride and NEFA concentrations were significantly elevated. In contrast, 5-HT did not affect the plasma cholesterol concentration, and it induced a decrease in bile acid concentrations. Increases in plasma glucose and insulin concentrations induced by 5-HT were attenuated by pre-treatment with Methysergide, a 5HTR 1, 2 and 7 antagonist. Additionally, decreased plasma bile acid concentrations induced by 5-HT were blocked by pre-treatment with Ketanserin, a 5HTR 2A antagonist. However, none of the 5HTR antagonists inhibited the increase in plasma triglyceride and NEFA levels induced by 5-HT. On the other hand, mRNA expressions of 5HTR1D and 1E were observed in the liver, pancreas and skeletal muscle. These results suggest that there are a number of differences in the physiological functions of peripheral 5-HT with respect to lipid metabolism between mice and sheep, though its effect on glucose metabolism appears to be similar between these species.

## Introduction

Serotonin (5-HT) is a monoaminergic neurotransmitter in the nervous system. In the periphery, 5-HT is produced from intestinal enterochromaffin cells by tryptophan hydroxylase (TPH) 1, which is the rate-limiting enzyme for 5-HT biosynthesis in this tissue. Another isoform of this enzyme is TPH2, which is found in the central nervous system [1]–[2]. Peripheral 5-HT acts as a hormone, affecting vasoconstriction, intestinal motility, primary haemostasis, liver repair and the control of T cell-mediated immunity [3]–[8]. The periphery contains approximately 98% of the body's 5-HT. Additionally, 5-HT is thought not to be able to pass the blood-brain barrier [9]–[10]. Thus, there are two independent 5-HT synthesis systems: one in the periphery and the other in the central nervous system.

It has been reported that increasing circulating concentrations of 5-HT induces hyperglycemia through the release of adrenaline from the adrenal gland in rats via action through the 5HTR1A, 2A or 7 [11]–[13]. Additionally, 5-HT stimulates glycogen synthesis at nanomolar concentrations but inhibits it at micromolar concentrations in hepatocytes by serotonergic mechanisms [14], and 5-HT enhances net hepatic glucose uptake under hyperglycemic and hyperinsulinemic conditions [15]. On the other hand, we have previously reported that peripheral 5-HT decreases plasma lipid levels via action through several 5HTRs in mice [16]. These results suggest that peripheral serotonin plays an important role in glucose and lipid metabolism.

It is well established that glucose and lipid metabolism in ruminants are substantially different to those of humans and rodents. For example, ruminants absorb little glucose and have no glucokinase activity in the liver, and nearly all of their glucose needs must be compensated for by gluconeogenesis [17]–[19]. Propionate is the principal source of carbon for glucose synthesis in liver, which meets 85–90% of the body glucose requirements in sheep [17], [20]. Sheep are also more resistant to insulin action compared with non-ruminants [18], [21]. Additionally, it has been shown that glucose, acetate, lactate and pyruvate are key raw materials in fatty acid biosynthesis in ruminants, and the rate of fatty acid synthesis from glucose in ruminant adipose tissue is very low [22]–[25]. Furthermore, some reports have suggested that there are differences in secretion and function of gastric hormones between monogastric species and ruminants. For instance, glucose is able to inhibit the secretion of ghrelin in non-ruminants, though short chain fatty acids decrease plasma ghrelin concentrations in wethers [26]–[28]. Ghrelin injection also increases plasma glucose concentrations in adult cows, while no hyperglycemic response to ghrelin is observed in suckling or pre-ruminant calves [29]. In non-ruminants, bile salts, amines, tastants and olfactants regulate 5-HT secretion from the enterochromaffin cells of the gastrointestinal tract [30]–[34]. Hence, it is likely that not only the secretion of 5-HT but also the response to 5-HT in ruminants may differ from that of monogastric animals, following the absorption of the products of rumen fermentation.

There are few reports of the effects of peripheral 5-HT in ruminant animals on glucose and lipid metabolism, even though it has been shown to be involved in those of mice. In the present study, in order to clarify the distinctive features of peripheral 5-HT in glucose and lipid metabolism of sheep, we explored the effect of 5-HT on the concentrations of plasma metabolites and insulin.

## Materials and Methods

### Ethics statement

The experiments were permitted by the Tohoku University and Iwate University Environmental & Safety Committee and conducted in accordance with the Guidelines for Animals Experimentation of Tohoku University and Iwate University, which have been sanctioned by the relevant committee of the Government of Japan based on the Declaration of Helsinki.

### Experimental animals

Six, crossbred (Corriedale × Suffolk) wethers, 1–3 years of age, weighing 47.2±7.8 kg were used for this study. Three animals were chosen at random from the six, and were used in 5HTR pre-treatment experiments. Animals were fed a maintenance diet of alfalfa hay cubes, and mixed orchard grass and reed canary grass hay at 1300 h each day. Water was available *ad libitum* throughout the experiment. In order to stably maintain the concentrations of plasma metabolites during the injection experiments, the animals were used for the experiments after fasting for 24 h. The weight of each wether did not change during the study. Experimental treatments were then conducted at weekly intervals. A catheter was inserted into the jugular vein of the animals and was filled with sodium citrate (0.13 mol/l) at 0900 h of the study day, and the injection study was performed at 1300 h.

### 5-HT and 5-HT receptor antagonist treatment experiments

5-HT (40 µg/kg body weight) (Sigma Chemical Co., St. Louis, MO) was dissolved in 5 ml phosphate buffered saline (PBS). Six animals were injected with a single dose of 5-HT into the jugular catheter. The dose used was determined according to previously published reports [35]–[37]. To eliminate the possibility of contamination by 5-HT in the catheter line, 10 ml PBS was flushed into the catheter after 5-HT injection and 5 ml of blood was disposed of prior to sampling. Blood samples were collected from the jugular catheter every 15 min from −30 to −15 min, every 3 min from 0 to 15 min, every 15 min from 30 to 60 min, every 30 min from 90 to 120 min, and every 60 min from 180 to 360 min, relative to the injection of 5-HT. Two ml of blood was obtained at each sampling time point. After a week, three of the experimental animals were used for the 5HTR antagonist pre-treatment experiments. Methysergide (antagonist for 5HTR1, 2 and 7; 40 µg/kg body weight; Sigma), SB-269970 (antagonist for 5HTR7; 70 µg/kg body weight; Tocris Bioscience, Ellisville, MO), SB-204070 (antagonist for 5HTR4; 40 µg/kg body weight; Sigma) and Ro 04-6790 (antagonist for 5HTR6; 30 µg/kg body weight; Sigma) were dissolved in PBS, respectively. Ketanserin (antagonist for 5HTR2A; 10 µg/kg body weight; Sigma) was dissolved in 0.1 M HCl, diluted with PBS. The final injection volume of all antagonists administrated was 5 ml. The doses of 5HTR antagonists were determined according to several published reports, bearing in mind the dose of 5-HT used [13],[16],[38]–[39]. These were administered 15 min prior to the injection of 5-HT. In addition to the blood sampling times for the 5-HT injection noted above, blood samples were also obtained from the jugular vein at 10 min and 5 min before the 5-HT injection for the antagonist experiments. The animals were also treated with PBS as control, and blood samples were taken between −30 and 120 minutes. Blood samples for the 5-HT assay were placed into a polyethylene tube containing EDTA (1 mg/ml) and centrifuged at 4500×g for 10 min at 4°C. Samples for the other analyses were placed into a polyethylene tube containing heparin (10 U/ml) and centrifuged at 9000×g for 10 min at 4°C. All samples were kept on ice until centrifugation. Following centrifugation, plasma was harvested and stored at −20°C until analysis.

### Biochemical Analysis

The plasma 5-HT and insulin concentrations were determined by commercial ELISA kit, which were purchased from Immunotech (Marseille, France) and Mercodia (Uppsala, Sweden), respectively. The plasma concentrations of glucose, TG, NEFA, cholesterol and bile acids were measured using a commercial colormetric kits (Wako, Osaka, Japan). All procedures were performed according to the respective manufacturer's instructions.

### Total Rna Preparation And Quantitative Real-Time Pcr Analysis

Total RNA were extracted from liver, pancreas, semitendinosus muscle and vastus intermedius muscle using Isogen II reagent according to the manufacturer's instructions (NIPPON GENE Inc., Tokyo, Japan). cDNA templates were synthesized with the Superscript III RT kit (Invitrogen, Co., Carlsabad, CA) using random primers. The quantitative real-time PCR was performed in duplicate using the Thermal Cycler Dice Real Time System Single (Takara Bio Inc., Siga, Japan) to assay specific 5HTR1A, 5HTR1B, 5HTR1D, 5HTR1E, 5HTR1F, 5HTR2A, 5HTR2B, 5HTR2C, 5HTR3A, 5HTR3B, 5HTR4, 5HTR5A and 5HTR7 mRNA levels. The sequences of primers and sizes of each PCR product are listed in Table 1. The typical reaction cycles consisted of an initial denaturation step 95°C for 30 sec followed by 40 cycles of denaturation at 95°C for 5 sec and annealing at 60 to 64°C for 30 sec. The resolution curve was measured at 95°C for 15 sec, 60°C for 15 sec and 95°C for 15 sec. Quantification of the RT-PCR products was normalized to the endogenous housekeeping gene (18s) expression.

**Table 1:** Primers used in quantitative real-time PCR analysis.

Genes		Primer sequence (5′-3′)	Product size (bp)	Tm °C
*5HTR 1A*	Forward	AATGGCCGCGTTGTACCAG	145	64
	Reverse	GGTGATGGCCCAGTATCTATCCAG		
*5HTR 1B*	Forward	TGATGCCCATCAGTACCATGT	71	60
	Reverse	CCAGAAGTCGCAGACCACCT		
*5HTR 1D*	Forward	ACCGCGCATCTCATCACAG	142	64
	Reverse	GTTCCAGGACACTATCGGCAAG		
*5HTR 1E*	Forward	AGTGTGGCTGTGAGACCCAAGA	105	64
	Reverse	CACGATCACGGCGGAGTTTA		
*5HTR 1F*	Forward	CAAGCAAGTAGGATTGCCAAGGA	101	64
	Reverse	TTTGCTAGCATGTACGGTGTGGA		
*5HTR 2A*	Forward	CCAGCCTTGGCCTACAAGTC	84	64
	Reverse	GTCATTATCTGTCGTCTTGCCATC		
*5HTR 2B*	Forward	GCCTCACCTACAGACATGGACAGA	117	64
	Reverse	AGGCTTTGTACCCATGCCAAAC		
*5HTR 2C*	Forward	GATTTGAACCCACGCCGAAG	134	64
	Reverse	AATGGGCACCACATGATCAGAA		
*5HTR 3A*	Forward	CACCTGCTGGCCAACTACAAGAA	101	64
	Reverse	ACGCTGAGGATGGCATAGACAA		
*5HTR 3B*	Forward	TGCAGAACAGCGCTGGAGA	83	64
	Reverse	CAGGCTCACCACGTAGACCAGA		
*5HTR 4*	Forward	CCTTGAATCTGGCCTTGCTG	117	64
	Reverse	CTTGAGCACTGCTTGGTCCTG		
*5HTR 5A*	Forward	GAAGATCTACAAGGCCGCCAAG	74	64
	Reverse	CGGTTTCGGATATGGGTGAGAC		
*5HTR 7*	Forward	CACTGCGGTAAGCCTAGTGATGAA	106	64
	Reverse	GCTTTGAACGGACACTGCTCTG		
*18s*	Forward	GCCCTATCAACTTTCGATGGTAGTC	113	64
	Reverse	CCTTGGATGTGGTAGCCGTTTC		

### Statistical Analysis

Values are reported as means ± S.D. Average concentrations of metabolites and hormones from −30 to 0 min before the 5-HT injection were taken to be basal values. Statistical significances between basal and subsequent concentrations of hormones and metabolites were determined using paired Student's t-test. To assess the effect of 5HTR antagonists, the incremental area was calculated by trapezoidal integration. Statistical significances between the PBS treatment and the antagonist treatments were determined using one-way ANOVA. P values less than 0.05 were considered to be statistically significant.

## Results

### Plasma 5-Ht Concentrations After Injection Of 5-Ht In Sheep

**Figure 1:**
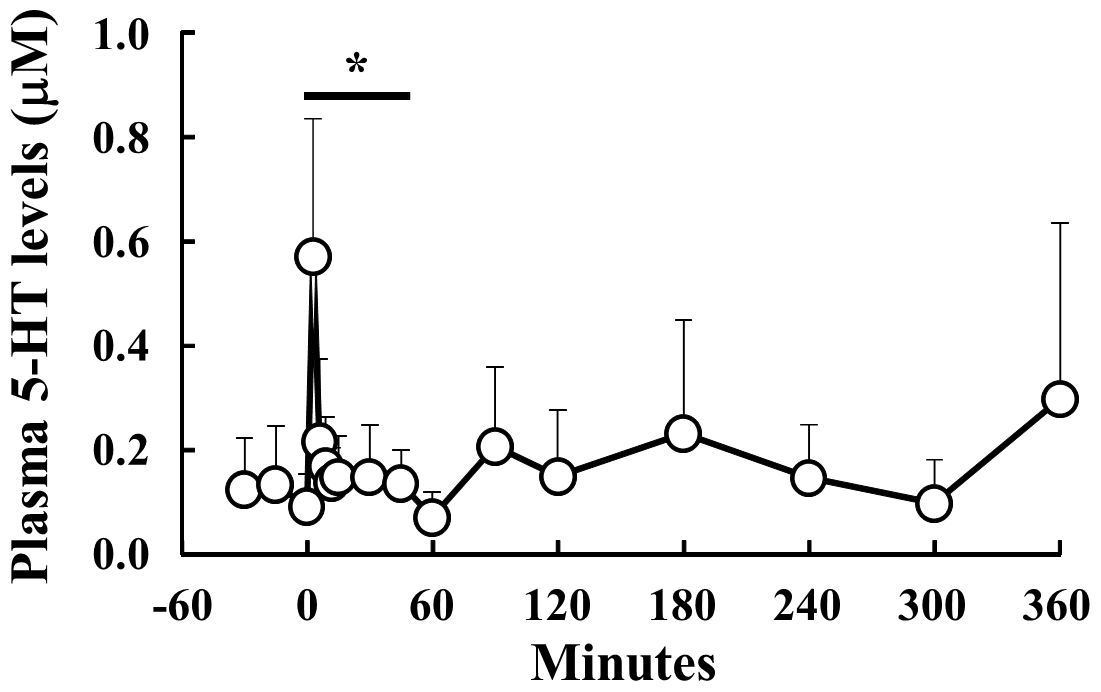
Plasma concentration of 5-HT following 5-HT injection. Blood samples were obtained from 6 sheep between −30 and 360 min relative to the intravenous injection of 5-HT (40 µg/kg body weight). The concentration of plasma 5-HT was measured. *: P<0.05 relative to the basal average values from −30 min to 0 min.

Relative to an intravenous injection of 5-HT, the plasma concentrations of 5-HT were measured between −30 min and 360 min (Figure 1). The average basal concentration of plasma 5-HT was approximately 0.1 µM before the injection of 5-HT. After the 5-HT injection, the plasma concentration of 5-HT reached a peak of 0.6 µM at 3 min, and sharply decreased thereafter. Concentrations returned to basal levels by 60 min.

### Effect Of Peripheral 5-Ht On Plasma Glucose Concentrations In Sheep

**Figure 2:**
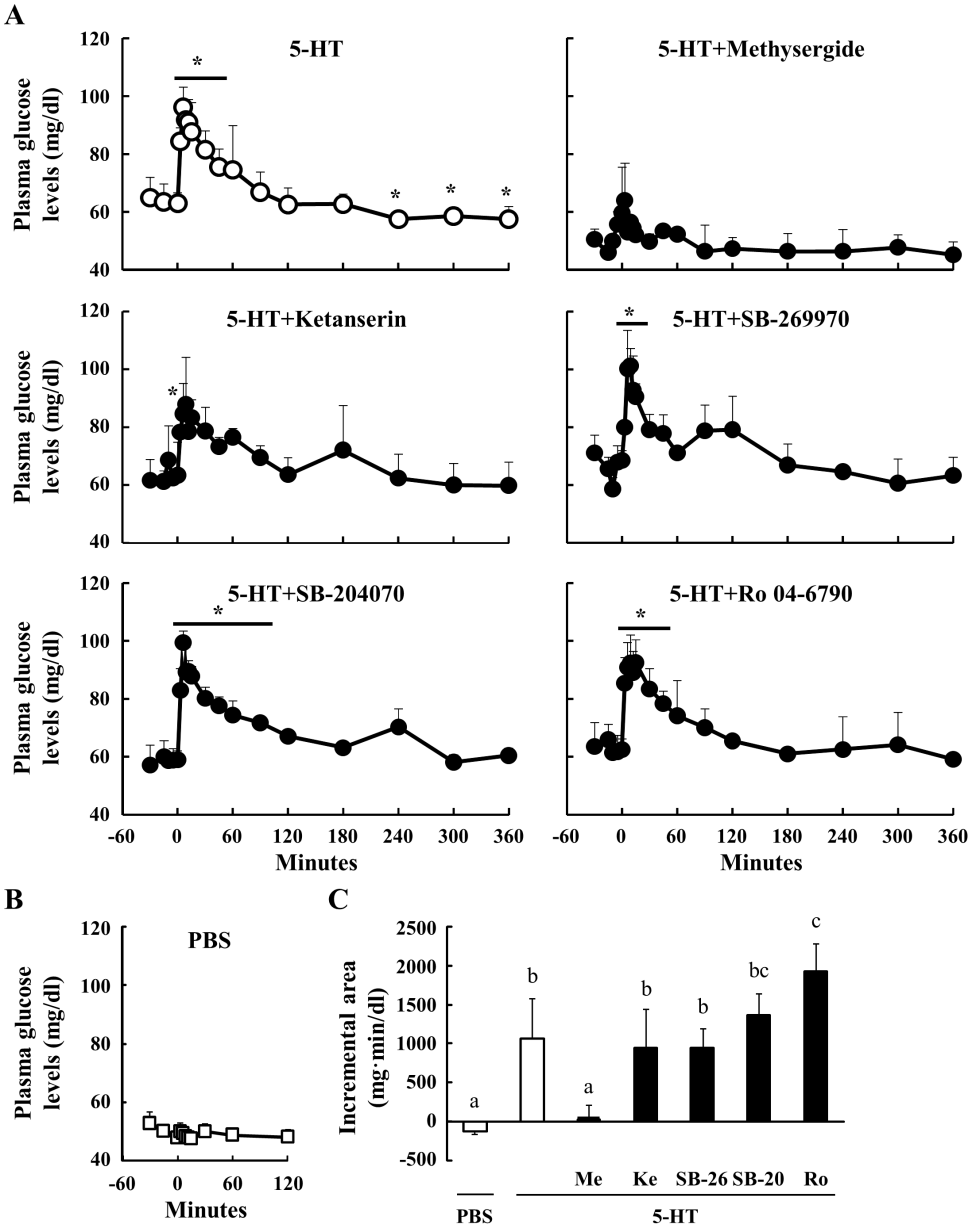
Effect of 5-HT injection on plasma glucose concentrations. Relative to an intravenous injection of 5-HT (40 µg/kg body weight) at 0 min, plasma samples were obtained from 6 sheep between −30 and 360 min (n = 6). Three sheep were injected through the jugular catheter with several 5HTR antagonists (n = 3): Methysergide (antagonist for 5HTR1, 2 and 7, 40 µg/kg body weight), Ketanserin (5HTR2A, 10 µg/kg body weight), SB-269970 (5HTR7, 70 µg/kg body weight), SB-204070 (5HTR4, 40 µg/kg body weight), and Ro 04-6790 (5HTR6, 30 µg/kg body weight), at 15 min before the injection of 5-HT. Plasma glucose concentrations were determined between −30 and 360 min (A). Plasma glucose levels after the injection of PBS were measured between −30 and 120 min (n = 3) (B). The incremental area between 0 and 60 min was calculated (C). *: P<0.05 relative to the basal average values from −30 to 0 min. Columns with a different letter are significantly different (P<0.05). Me: Methysergide; Ke: Ketanserin; SB-26: SB-269970; SB-20: SB-204070; Ro: Ro 04-6790.

Following 5-HT injection, the plasma glucose concentration was significantly elevated from 3 min to 45 min, reached a peak at 6 min, gradually returned to basal levels, and was slightly lower than basal levels between 240 min to 360 min (Figure 2a). No significant variations in circulating glucose concentrations were observed before the 5-HT injection. In order to determine what kind of 5HTRs were related to the hyperglycemia induced by 5-HT, sheep were pre-treated with several 5HTR antagonists: Methysergide (5HTR1, 2 and 7), Ketanserin (5HTR2A), SB-269970 (5HTR7), SB-204070 (5HTR4) or Ro 04-6790 (5HTR6) at 15 min before 5-HT injection. Pre-treatments of all 5HTR antagonists did not influence the concentration of plasma metabolites. Pre-treatment with Methysergide completely inhibited the hyperglycemia induced by 5-HT. However, pretreatment with the other antagonists did not significantly affect hyperglycemia. Additionally, plasma glucose concentrations were not affected between −30 and 120 min by the injection of PBS (Figure 2b). The incremental areas of glucose concentrations following 5-HT injection alone and following pre-treatment with Ketanserin, SB-269970, SB-204070 and Ro 04-6790 were significantly greater than that of PBS injection (Figure 2c). In contrast, the incremental area following pre-treatment with Methysergide was not significantly increased compared with that of PBS injection. These data suggest that peripheral 5-HT increases plasma glucose concentrations through 5HTR1.

### Effect Of Peripheral 5-Ht On Plasma Insulin Concentrations In Sheep

**Figure 3:**
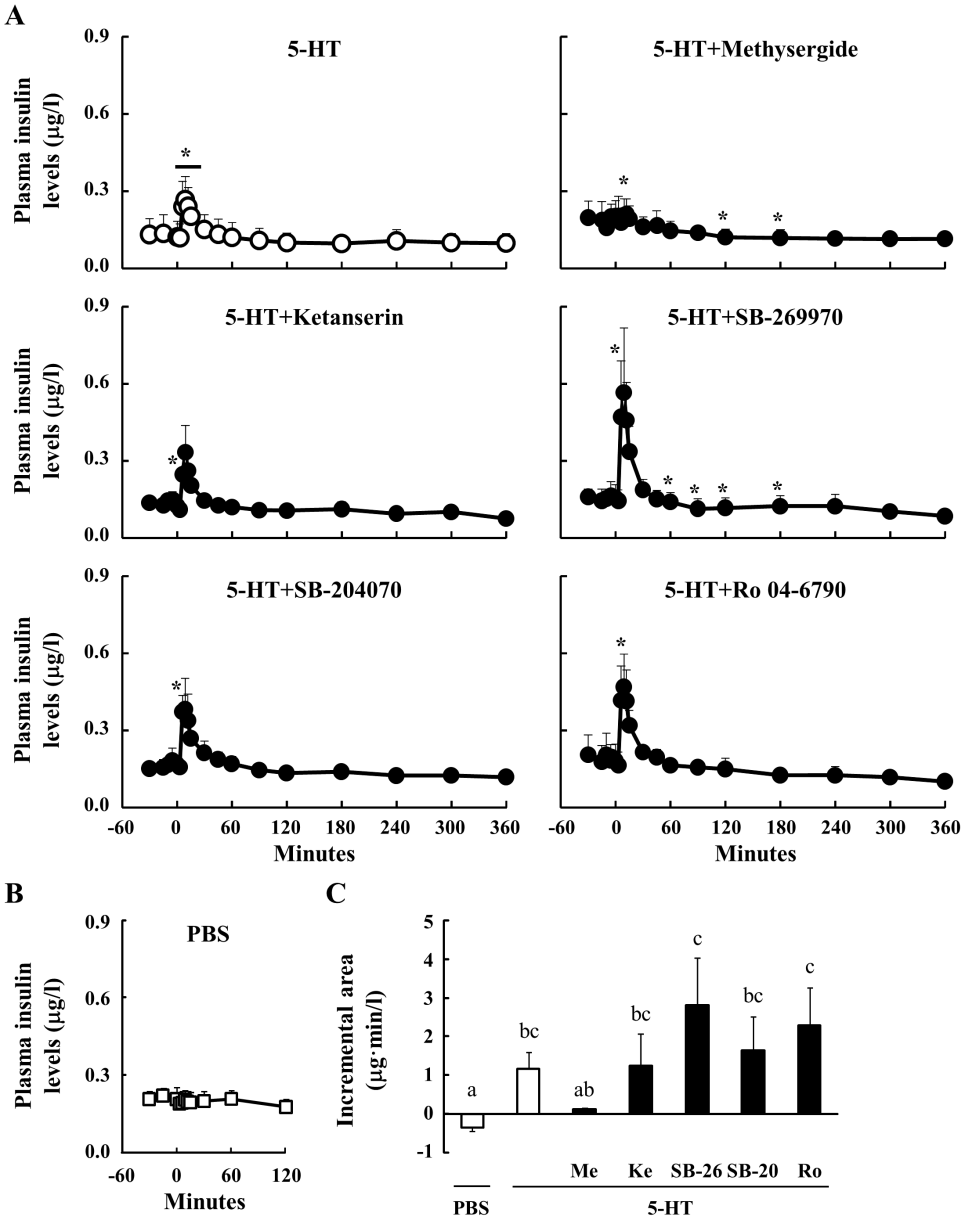
Effect of 5-HT injection on plasma insulin concentrations. Relative to an intravenous injection of 5-HT (40 µg/kg body weight) at 0 min, plasma samples were obtained from 6 sheep between −30 and 360 min (n = 6). Three sheep were injected through the jugular vein with several 5HTR antagonists (n = 3): Methysergide (antagonist for 5HTR1, 2 and 7, 40 µg/kg body weight), Ketanserin (5HTR2A, 10 µg/kg body weight), SB-269970 (5HTR7, 70 µg/kg body weight), SB-204070 (5HTR4, 40 µg/kg body weight), and Ro 04-6790 (5HTR6, 30 µg/kg body weight), at 15 min before the injection of 5-HT. Plasma insulin concentrations were determined between −30 and 360 min (A). Plasma insulin levels after the injection of PBS were measured between −30 and 120 min (n = 3) (B). The incremental area between 3 and 15 min was calculated (C). *: P<0.05 relative to the basal average values from −30 to 0 min. Columns with a different letter are significantly different (P<0.05). Me: Methysergide; Ke: Ketanserin; SB-26: SB-269970; SB-20: SB-204070; Ro: Ro 04-6790.

After the 5-HT injection alone, plasma insulin concentrations were significantly increased between 6 and 15 min post injection. Levels were maximal at 9 min and were not significantly different from basal values after 30 min (Figure 3a). The concentration of plasma insulin before the injection of 5-HT was constant. Pre-treatment with Methysergide inhibited the increase in plasma insulin levels caused by administration of 5-HT in sheep, and levels were significantly lower than basal values between 120 and 180 min after the 5-HT injection. Pre-injection with Ketanserin, SB-204070 and Ro 04-6790 did not block the elevation in plasma insulin concentrations induced by 5-HT. However, in sheep pre-treated with SB-269970, hypoinsulinemia was observed between 60 and 180 min after the 5-HT administration. On the other hand, the administration of PBS did not affect plasma insulin concentrations (Figure 3b). The incremental area for Methysergide was not significantly different to that for the PBS injection (Figure 3c). In contrast, insulin levels for the other antagonist pretreatments were significantly increased compared with that for PBS injection. These results suggest that peripheral 5-HT induces hyperinsulinemia through the 5HTR1.

### Effect Of Peripheral 5-Ht On The Plasma Triglyceride Concentrations In Sheep

**Figure 4:**
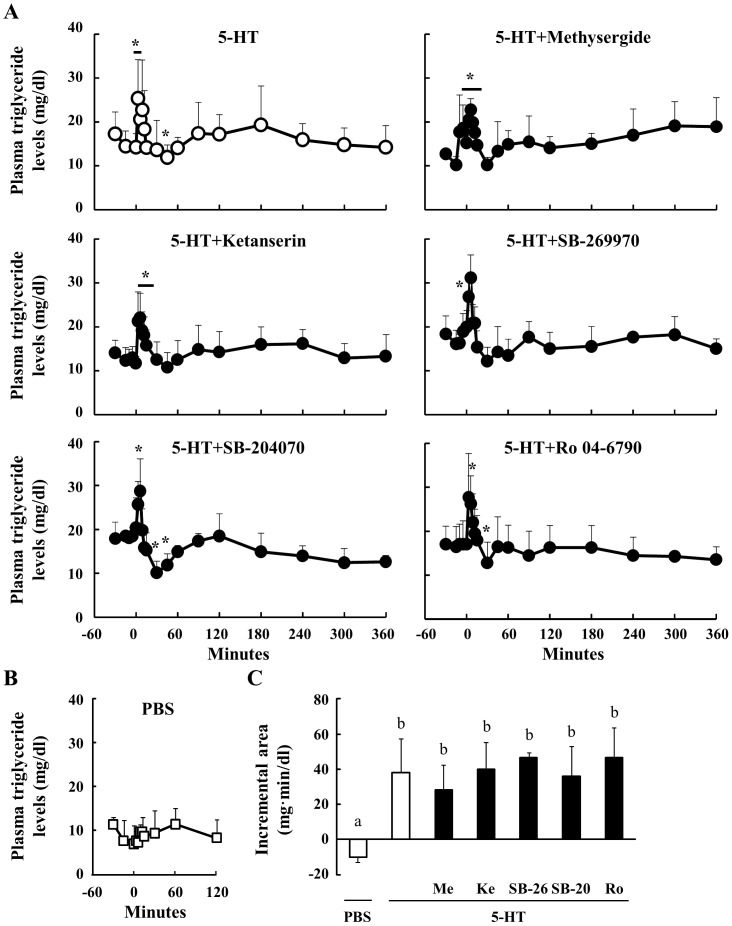
Effect of 5-HT injection on plasma triglyceride concentrations. Relative to an intravenous injection of 5-HT (40 µg/kg body weight) at 0 min, plasma samples were obtained from 6 sheep between −30 and 360 min (n = 6). Three sheep were injected through the jugular vein with several 5HTR antagonists (n = 3): Methysergide (antagonist for 5HTR1, 2 and 7, 40 µg/kg body weight), Ketanserin (5HTR2A, 10 µg/kg body weight), SB-269970 (5HTR7, 70 µg/kg body weight), SB-204070 (5HTR4, 40 µg/kg body weight), and Ro 04-6790 (5HTR6, 30 µg/kg body weight), at 15 min before the injection of 5-HT. Plasma triglyceride concentrations were determined between −30 and 360 min (A). Plasma triglyceride levels after the injection of PBS were measured between −30 and 120 min (n = 3) (B). The incremental area between 0 and 6 min was calculated (C). *: P<0.05 relative to the basal average values from −30 to 0 min. Columns with a different letter are significantly different (P<0.05). Me: Methysergide; Ke: Ketanserin; SB-26: SB-269970; SB-20: SB-204070; Ro: Ro 04-6790.

5-HT significantly induced an elevation in plasma triglyceride levels between 3 and 6 min after the injection. However, concentrations were significantly decreased relative to basal values by 45 min after the 5-HT injection (Figure 4a). Before the administration of 5-HT, plasma triglyceride levels were stable. Pre-injection with Methysergide, Ketanserin and SB-269970 did not affect the hypertriglyceridemia induced by 5-HT, but they did appear to prevent the significant reduction in plasma triglyceride concentrations observed at 45 min after the 5-HT injection. Pre-treatment with SB-204070 and Ro 04-6790 did not affect the profile of plasma triglycerides relative to 5-HT alone. Additionally, no significant effects of the PBS injection on plasma triglyceride concentrations were observed (Figure 4b). The incremental area of all groups was greater than that of the PBS treatment (Figure 4c). These results indicate that the hypertriglyceridemia induced by 5-HT does not depend on 5HTR1, 2, 4, 6 or 7, and that 5HTR1, 2 and 7 may be related to the hypotriglyceridemia induced by peripheral 5-HT.

### Effect Of Peripheral 5-Ht On Plasma Nefa And Cholesterol Concentrations In Sheep

**Figure 5:**
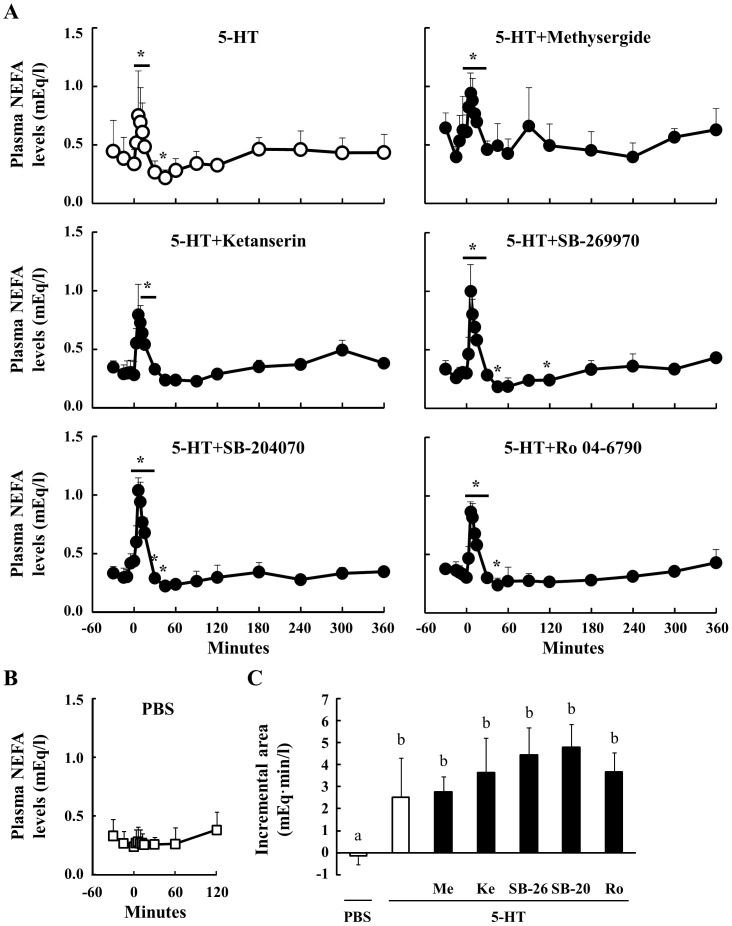
Effect of 5-HT injection on plasma NEFA concentrations. Relative to an intravenous injection of 5-HT (40 µg/kg body weight) at 0 min, plasma samples were obtained from 6 sheep between −30 and 360 min (n = 6). Three sheep were injected through the jugular catheter with several 5HTR antagonists (n = 3): Methysergide (antagonist for 5HTR1, 2 and 7, 40 µg/kg body weight), Ketanserin (5HTR2A, 10 µg/kg body weight), SB-269970 (5HTR7, 70 µg/kg body weight), SB-204070 (5HTR4, 40 µg/kg body weight), and Ro 04-6790 (5HTR6, 30 µg/kg body weight), at 15 min before the injection of 5-HT. Plasma NEFA concentrations were determined between −30 and 360 min (A). Plasma NEFA levels after the injection of PBS were measured between −30 and 120 min (n = 3) (B). The incremental area between 0 and 12 min was calculated (C). *: P<0.05 relative to the basal average values from −30 to 0 min. Columns with a different letter are significantly different (P<0.05). Me: Methysergide; Ke: Ketanserin; SB-26: SB-269970; SB-20: SB-204070; Ro: Ro 04-6790.

Plasma NEFA concentrations were significantly increased between 6 and 15 min after the 5-HT injection, but were decreased by 45 min, relative to the baseline levels (Figure 5a). The concentration of plasma NEFA was constant before the injection of 5-HT. Pre-administration with the antagonists did not attenuate the elevation of plasma NEFA concentrations in the minutes immediately following the 5-HT injection. However, pre-treatment with Methysergide and Ketanserin prevented the reduction in plasma NEFA concentrations at 45 min after 5-HT treatment. Additionally, the injection of PBS did not affect plasma NEFA concentrations (Figure 5b). Moreover, none of the 5HTR antagonists affected the incremental area under the NEFA concentrations induced by 5-HT (Figure 5c).

**Figure 6:**
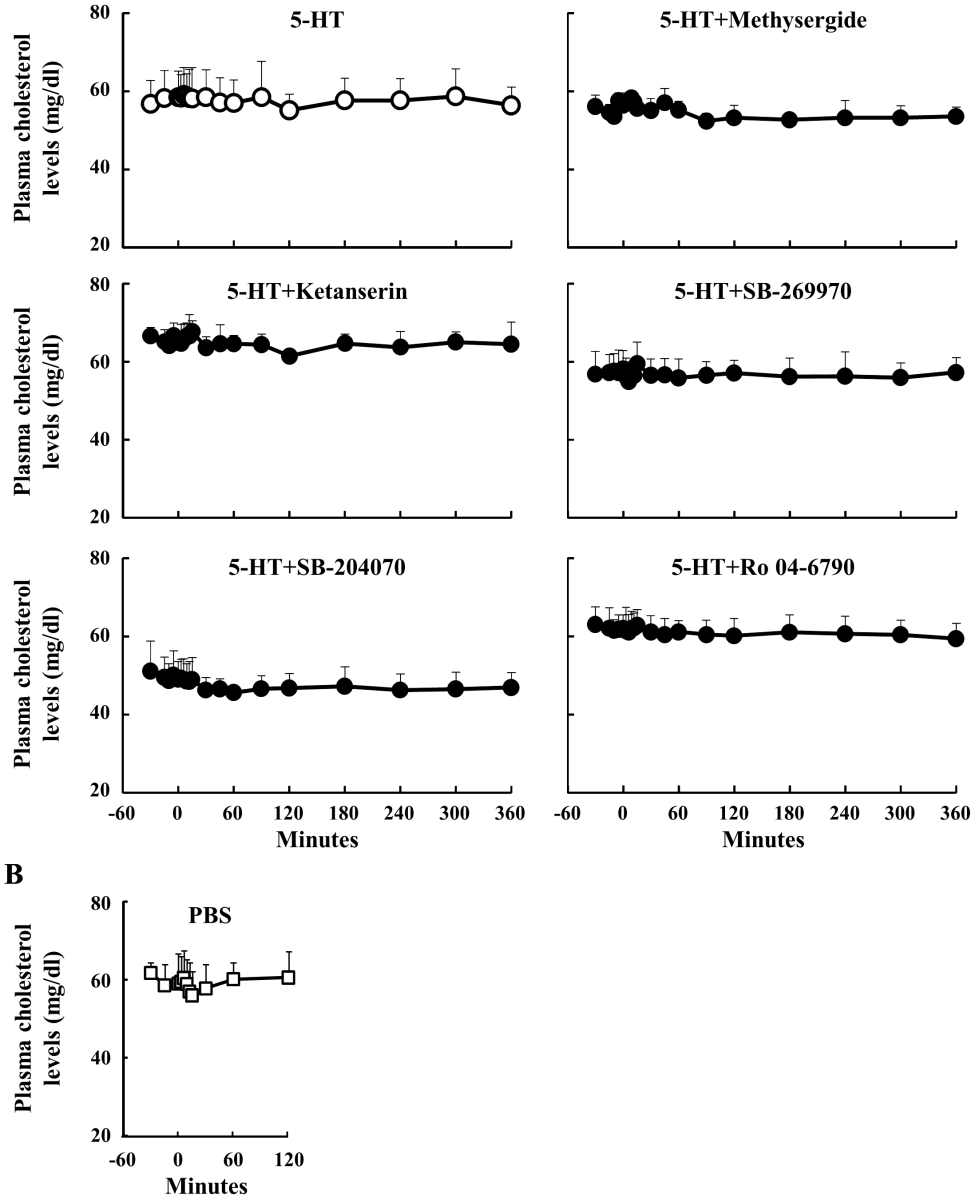
Effect of 5-HT injection on plasma cholesterol concentrations. Relative to an intravenous injection of 5-HT (40 µg/kg body weight) at 0 min, plasma samples were obtained from 6 sheep between −30 and 360 min (n = 6). Three sheep were injected through the jugular catheter with several 5HTR antagonists (n = 3): Methysergide (antagonist for 5HTR1, 2 and 7, 40 µg/kg body weight), Ketanserin (5HTR2A, 10 µg/kg body weight), SB-269970 (5HTR7, 70 µg/kg body weight), SB-204070 (5HTR4, 40 µg/kg body weight), and Ro 04-6790 (5HTR6, 30 µg/kg body weight), at 15 min before the injection of 5-HT. Plasma cholesterol concentrations were determined between −30 and 360 min (A). Plasma cholesterol levels after the injection of PBS were measured between −30 and 120 min (n = 3) (B). *: P<0.05 relative to basal average values from −30 min to 0 min.

We have reported previously that peripheral 5-HT substantially decreases plasma cholesterol concentrations in mice [16]. However, 5-HT and PBS injections did not affect the plasma cholesterol concentration in sheep in the present experiment (Figure 6a, 6b). Additionally, none of the 5-HT antagonists affected the level of plasma cholesterol.

### Effect Of Peripheral 5-Ht On The Concentration Of Plasma Bile Acids In Sheep

**Figure 7:**
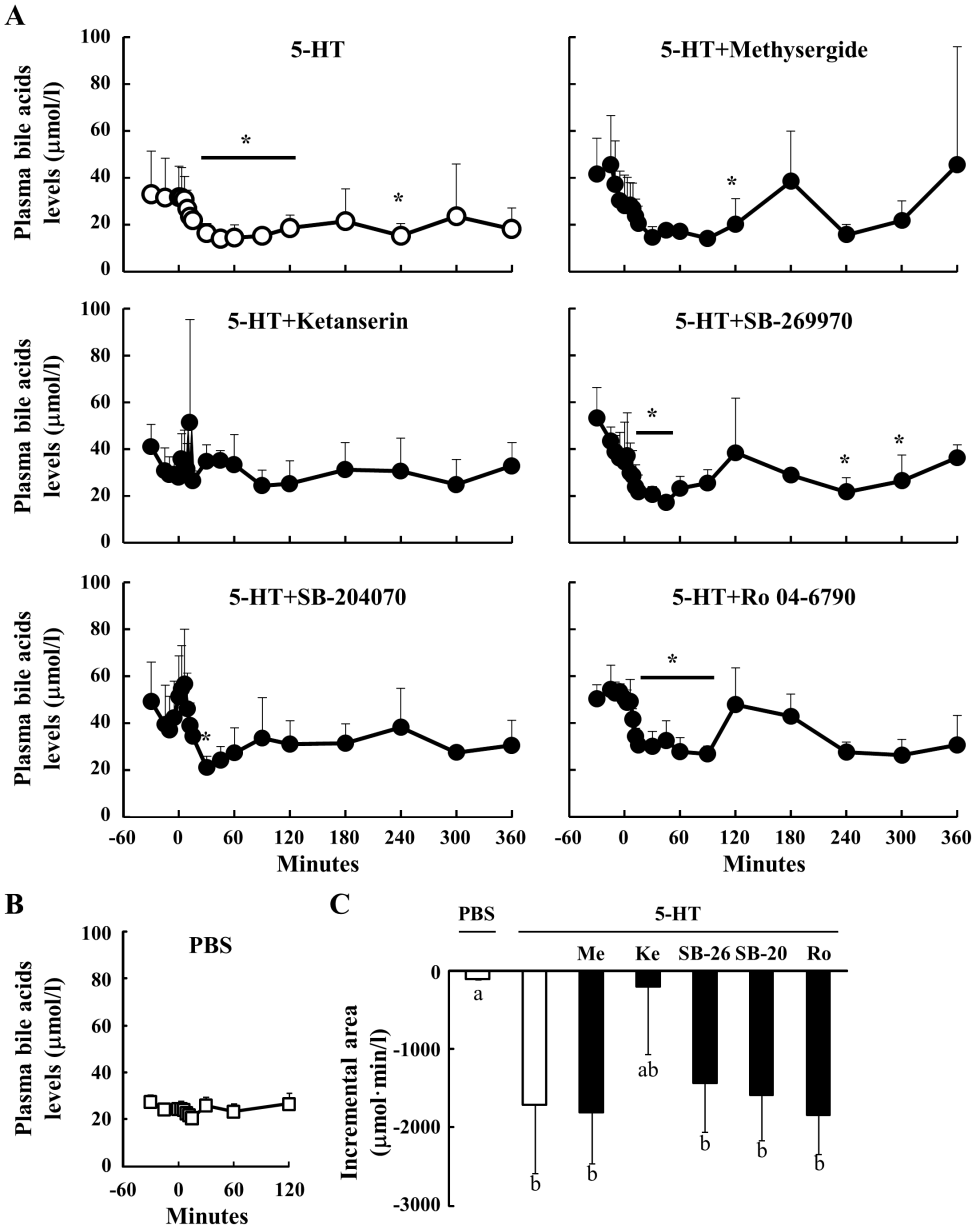
Effect of 5-HT injection on plasma bile acid concentrations. Relative to an intravenous injection of 5-HT (40 µg/kg body weight) at 0 min, plasma samples were obtained from 6 sheep between −30 and 360 min (n = 6). Three sheep were injected through the jugular vein with several 5HTR antagonists (n = 3): Methysergide (antagonist for 5HTR1, 2 and 7, 40 µg/kg body weight), Ketanserin (5HTR2A, 10 µg/kg body weight), SB-269970 (5HTR7, 70 µg/kg body weight), SB-204070 (5HTR4, 40 µg/kg body weight), and Ro 04-6790 (5HTR6, 30 µg/kg body weight), at 15 min before the injection of 5-HT. Plasma bile acid concentrations were determined between −30 and 360 min (A). Plasma bile acid levels after the injection of PBS were measured between −30 and 120 min (n = 3) (B). The incremental area between 30 and 120 min was calculated (C). *: P<0.05 relative to the basal average values from −30 to 0 min. Columns with a different letter are significantly different (P<0.05). Me: Methysergide; Ke: Ketanserin; SB-26: SB-269970; SB-20: SB-204070; Ro: Ro 04-6790.

The concentration of plasma bile acids was significantly lower following the 5-HT injection relative to basal levels. Concentrations returned to the baseline after 300 min (Figure 7a). Prior to the injection of 5-HT, no significant differences in plasma bile acid concentrations were observed. The concentration of bile acids was decreased following 5-HT and pre-treatment with Methysergide, SB-269970, SB-204070 and Ro 04-6790. In contrast, the Ketanserin attenuated the reduction in the concentration of plasma bile acids induced by 5-HT. Additionally, plasma bile acid concentrations were not decreased by the administration of PBS (Figure 7b). Although the incremental area of bile acid concentrations following pre-treatment with Ketanserin was similar to that of PBS, those following 5-HT and pretreatment with the other antagonists were significantly lower than that of PBS (Figure 7c). These results suggest that 5-HT induces a decrease in the plasma concentrations of plasma bile acids through the 5HTR2A.

### Mrna Expressions Of 5htrs In The Liver, Pancreas And Skeletal Muscle

**Figure 8:**
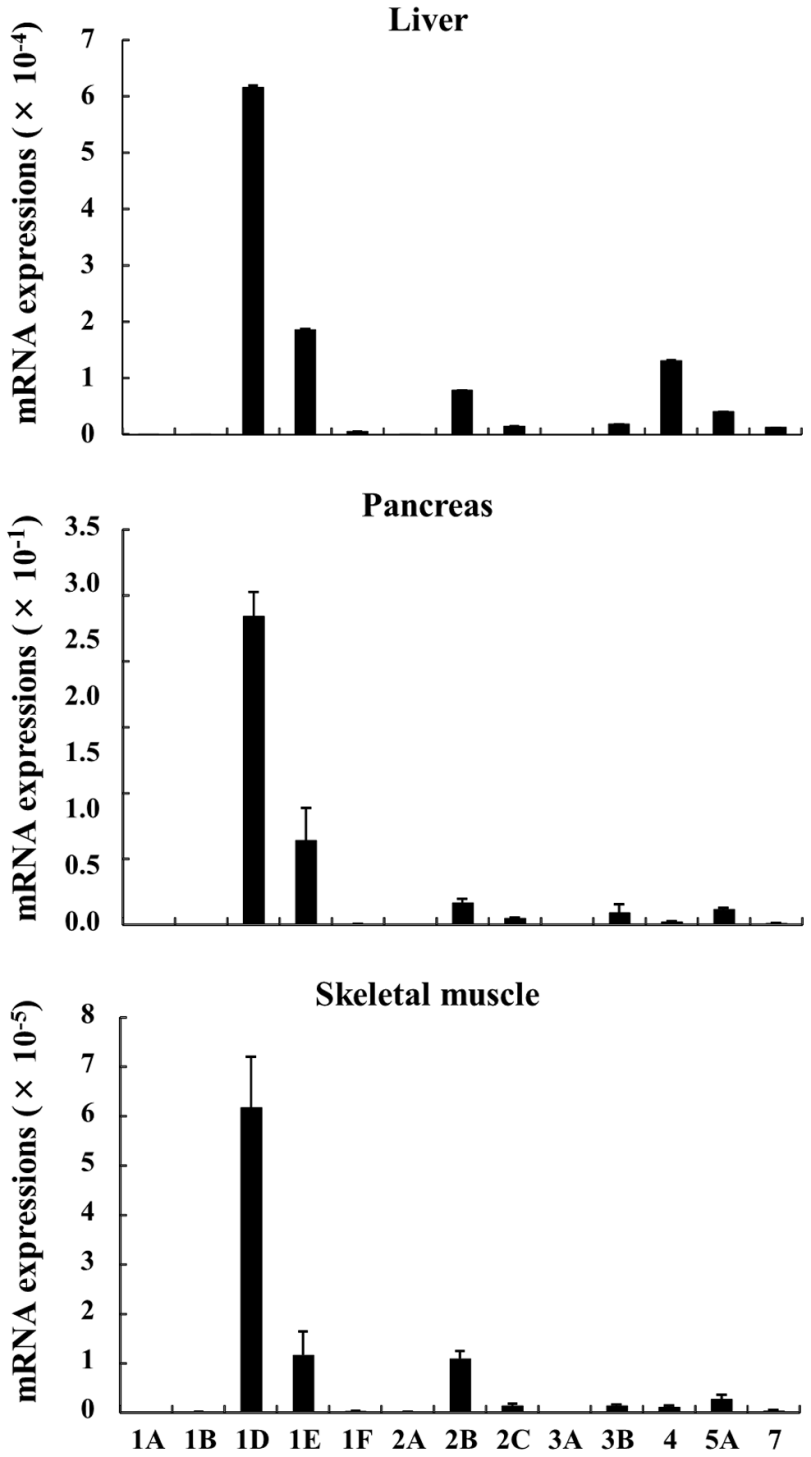
mRNA expressions of 5HTRs in the liver, pancreas and skeletal muscle. Liver, pancreas and skeletal muscle tissues were obtained from 3 sheep. After total RNA from each tissue was extracted, 5HTRs mRNA expressions were measured by real-time PCR analysis.

To identify primary forms of 5HTRs in metabolically active tissues, we investigated the mRNA expression of 5HTRs in the liver, pancreas and skeletal muscle. In these tissues, 5HTR1D, 1E, 2B and 5A were the main forms expressed in these tissues; this was particularly the case for 5HTR1D (Figure 8). 5HTR4 was expressed only in the liver.

## Discussion

There are two independent 5-HT systems, one in the brain and the other in the periphery. In humans and rodents, 5-HT regulates glucose and lipid metabolism through several 5HTRs and the 5-HT transporter (SERT) [11]–[16],[40]–[41]. However, we are not aware of any reports on the function of peripheral 5-HT in ruminant animals. The main objective of this study was to clarify the role of peripheral 5-HT in sheep by investigating the effect of intravenous 5-HT injection on the concentrations of metabolites related to glucose and lipid metabolism in the circulation.

5-HT is known to be associated with glucose metabolism, mainly because of its regulation of the secretion of insulin in pancreatic β cells. The insulin secretion induced by glucose is inhibited by 5-HT in rat islet of Langerhans incubations *in vitro* [41]. Another report demonstrates that 5-HT regulates insulin secretion by serotonylation of GTPase within the pancreatic β-cells [40]. Additionally, 5-HT directly controls the uptake of glucose into the peripheral tissues, including the liver and skeletal muscles [42]–[43]. 5-HT enhances net hepatic glucose uptake under hyperglycemic and hyperinsulinemic conditions [15], and stimulates glycogen synthesis at nanomolar concentrations, but inhibits it at micromolar concentrations by serotonergic mechanisms in hepatocytes [14]. Moreover, we previously reported that 5-HT induced the elevation of plasma glucose and insulin concentrations through different 5HTRs in mice, and that hyperglycemia after the injection of 5-HT was induced by repressing glucose uptake into the tissues [16].

Considering the data in this report, there appear to be a number of differences in 5-HT action between ruminants and nonruminants; the effect of 5-HT on plasma metabolite and insulin concentrations in sheep and mice is summarized in Table 2. In this study, plasma glucose and insulin concentrations were increased following the 5-HT injection, as seen in mice. Additionally, elevated plasma glucose and insulin levels in sheep were prevented following pre-treatment with Methysergide. In contrast, pre-injection with Ketanserin and SB-269970 prevented the elevation of plasma glucose concentrations induced by 5-HT in mice, but not sheep. Moreover, high mRNA expressions of 5HTR1D and 1E were observed, but 5HTR2A and 7 mRNA expressions were lower in the liver and pancreas of sheep. These data indicate that intravenous 5-HT injection induces hyperglycemia and hyperinsulinemia only through the 5HTR1 in sheep. It is well established that the digestion, absorption, utilization and production of glucose in ruminants differ greatly from those of in monogastric animals [17]–[21]. However, the recovery to basal levels of plasma insulin after the 5-HT administration was faster than that of glucose in both sheep and mice, though an increase in plasma insulin concentrations was observed after the elevation of plasma glucose level following 5-HT injection [16]. In addition, 5-HT appears to directly regulate insulin secretion from pancreatic β cells [40]–[41]. Taken together, these data suggest that the elevation of plasma glucose and insulin concentrations induced by 5-HT may have independent pathways in sheep and mice.

**Table 2:** Comparison of the effects of several 5-HT receptor antagonists on the plasma concentrations of metabolites and insulin, following 5-HT injection in sheep and mice.

	Sheep						Mouse^a^			
Plasma contents	5-HT^b^	Me	Ke	SB-26	SB-20	Ro	5-HT	Me	Ke	SB-26
Glucose	I	**+**	−	−	−	−	I	+	+	+
Insulin	I	+	−	−	−	−	I	+	−	−
Triglyceride	I	−	−	−	−	−	D	−	−	+
NEFA	I	−	−	−	−	−	D	−	+	−
Cholesterol	N	−	−	−	−	−	D	+	−	−
Bile acids	D	−	+	−	−	−	I	+	+	−

a Murine data published previously [Ref. 16].

b 5-HT: serotonin; Me: methysergide; Ke: ketanserin; SB-26: SB-269970; SB-20: SB-204070; Ro: Ro 04-6790; I: increase; D: decrease; N: no change; +: effective in blocking the effect of 5-HT; −: ineffective in blocking the effect of 5-HT.

In mice, plasma triglyceride, NEFA and cholesterol concentrations are decreased by peripheral 5-HT through 5HTR1, 2A and 7 (Table 2) [16]. In sheep, 5-HT administration resulted in an increase in plasma triglyceride and NEFA levels, but it did not affect plasma cholesterol levels in this study. Sheep are characterized by low concentrations of plasma VLDL and a high ratio of plasma HDL, compared with the mouse [44]–[46]. In addition, the concentration of each plasma lipid is also controlled by regulating the homeostatic uptake and release of lipids in the various tissues by 5-HT through several 5HTRs [47]. Therefore, the difference in plasma lipid concentrations induced by peripheral 5-HT may be attributable to differences in the concentrations and the ratio of each lipoprotein between sheep and mice. Differences in their responses to 5-HT in terms of plasma lipid levels may be caused by different expressions of 5HTRs in the various tissues, as the metabolism and functions of each tissue in ruminant animals differ from those of in monogastric animals.

In mice, plasma triglyceride and NEFA concentrations were decreased by peripheral 5-HT injection; these effects were blocked by SB-269970 and Ketanserin, respectively (Table 2). The present study revealed that the effect of 5-HT on the plasma triglyceride and NEFA concentrations in sheep was opposite to that seen in mice. However, the decrease in plasma triglyceride and NEFA concentrations relative to basal values was also observed in sheep, as for mice after each peak. The reduced plasma triglyceride level at 45 min after 5-HT injection in sheep was inhibited by three of the 5HTR antagonists: Methysergide, Ketanserin and SB-269970. In addition, decreased plasma NEFA concentrations at 45 min after injection of 5-HT was blocked by Methysergide and Ketanserin. Thus, there is a similar inclination between sheep and mice in terms of the decrease of plasma triglyceride and NEFA concentrations induced by 5-HT, although not identical results of 5-HT treatment. On the other hand, 5-HT did not appear to affect the metabolism of cholesterol in sheep.

After 5-HT injection, the concentration of plasma bile acids was decreased in sheep (Table 2). In contrast, the concentration of bile acids in plasma in mice is increased between 30 and 90 min after a 5-HT injection, due to an elevated re-absorption in the ileum [16] and an induced excretion from the gallbladder [48]. Additionally, after three days of ligation of the bile duct, mice lacking peripheral 5-HT display higher levels of plasma bile salts than wild type mice, as Ostα and Ostβ, the bile salt re-absorption transporters, are up-regulated in the kidneys, along with a decrease in urinary bile salt excretion [49]. Besides this, in ruminants, the excretion of bile from the gallbladder occurs following the absorption of lipids [50]. Thus, rapid excretion of bile acids cannot occur following an increase in the plasma 5-HT concentration in sheep. Therefore, the decreased concentrations of plasma bile acids in sheep may be induced by 5-HT treatment without the excretion of bile from the gallbladder, in contrast to what is seen in rodents. In mice, Methysergide and Ketanserin antagonized the elevation in the concentration of plasma bile acids after 5-HT injection (Table 2). Additionally, Ketanserin administration to sheep demonstrated that 5-HT induced a decrease in the concentration of plasma bile acids through 5HTR2A. These data indicate that 5-HT has the opposite effects on the concentration of plasma bile acids in sheep and mice, but through the same 5HTR2A. However, the expression of 5HTR2A was lower than other 5HTRs in the liver of sheep. The non-hepatic tissues are probably concerned with a decrease in the concentration of bile acids in the circulation by 5-HT, though it is known that the liver is greatly concerned with bile acids metabolism.

Fifteen 5-HT receptors have been identified in the mouse. They have been divided into seven distinct classes, 5HTR1 to 7, largely on the basis of their structure and operational characteristics. 5HTRs belong to the G-protein-coupled receptor (GPCR) subfamily, except for 5HTR3, which is a ligand-gated ion channel, and all are found both in the central nervous system and in many peripheral tissues. 5-HT is thought to exert its effects through these membrane bound receptors [51]. However, there are few reports on the distribution of 5HTRs in sheep. Accordingly, in order to investigate the physiological role of 5-HT, the localization of 5HTRs in ruminants needs to be established.

Ruminants have a distinct metabolism with respect to their utilization and production of glucose and fatty acids [17]–[25]. Therefore, it is likely that 5-HT may also have different functions with regard to glucose and lipid metabolism in ruminants. Here, we have investigated the effects of 5-HT injection on glucose and lipid metabolisms in sheep. In conclusion, we note that peripheral 5-HT affects the metabolism of glucose in sheep through the same mechanism and 5HTRs as mice, but that it has different functions with respect to lipid metabolism between sheep and mice.
